# Sustainable ecofriendly phytoextract mediated one pot green recovery of chitosan

**DOI:** 10.1038/s41598-019-50133-z

**Published:** 2019-09-25

**Authors:** Judy Gopal, Manikandan Muthu, Thirumalai Dhakshanamurthy, Ki Jun Kim, Nazim Hasan, Seong Jung Kwon, Sechul Chun

**Affiliations:** 1 Department of Environmental Health Science, Konkuk University, Seoul, 05029 South Korea; 2 Department of Chemistry, Thiruvalluvar University, Vellore, Tamil Nadu 632115 India; 3 Department of Chemistry, Konkuk University, Gwangjin-gu, Seoul Korea; 4 Department of Chemistry, Faculty of Science, Jazan University, Jazan, P.O. Box 114 Saudi Arabia

**Keywords:** Biopolymers in vivo, Nanoparticles

## Abstract

Chitin and chitosan are biopolymers that have diverse applications in medicine, agriculture, food, cosmetics, pharmaceuticals, wastewater treatment and textiles. With bio-origins, they easily blend with biological systems and show exemplified compatibility which is mandatory when it comes to biomedical research. Chitin and chitosan are ecofriendly, however the processes that are used to recover them aren’t ecofriendly. The focus of this work is to attempt an ecofriendly, sustainable phytomediated one pot recovery of chitosan from commercial chitin and from crab and shrimp shells and squid pen solid wastes. Graviola extracts have been employed, given the fact file that their active ingredients acetogenins actively interact with chitin in insects (resulting in its application as an insecticide). With that as the core idea, the graviola extracts were chosen for orchestrating chitin recovery and a possible chitin to chitosan transformation. The results confirm that graviola extracts did succeed in recovery of chitosan nanofibers from commercial chitin flakes and recovery of chitosan particles directly from solid marine wastes of crab, shrimp and squids. This is a first time report of a phyto-extract mediated novel chitosan synthesis method.

## Introduction

Chitin is nature’s benevolence, as part of the exoskeleton of mollusks^1^, Arthropods^2,3^, in the cell wall of mushrooms^4^ and in body structures of corals^5^ and sponges^6–9^ and algae^10,11^. It is also the cell wall component of fungi and yeast^12^. Chitin has also been discovered in fossils^13^. Being the second most common polysaccharide on the planet after cellulose, chitin is a worthwhile contribution from the animal kingdom as much as cellulose is from the plant kingdom. Chemically, chitin is a linear polymer consisting mainly of β-(1 → 4)-linked 2-acetamido-2-deoxy-β-D-glucopyranose units and partially of β-(1 → 4)-linked 2-amino-2-deoxy-β-D-glucopyranose^14^. The estimated production of chitin is reported to be around 1010–1012 tonnes per year^15^.

In nature, chitin occurs in two allomorphs, α and β-forms and additionally as γ-chitin (a combination of α and β structures)^16^. The predominant of these three is reported to be the alpha form^17^. Depending on the source, α-Chitin is by far the abundant and most frequently isolated form. It is obtained from the exoskeleton of crustaceans, particularly from shrimps and crabs. β-Chitin is obtained from squid pens and γ-chitin from fungi, yeast and cocoon of moths^18,19^. The percentages of chitin in crab and shrimp shells is 20% (% of dry weight) and in squid pens 31%, these contents mark the highest values occurring in commonly accessible marine products. The other less commonly available sources include, cicada sloughs (36%), silkworm chrysalides (20%) and cyst shells (29–34%)^6^. Crab and shrimp shells and krills remain the most utilized sources for chitin recovery. A derivative of chitin, chitosan is a linear polysaccharide of (1–4)-linked 2-amino-2-deoxy-β-D-glucopyranose monomers. It is made by deacetylation of the extracted chitin. The chitin deacetylation using a strong alkaline medium has been considered as the main method for providing chitosan^20,21^. Whenever the degree of deacetylation (DDA) reaches higher than approximately 90%, chitosan becomes soluble in acidic solutions^22,23^. However, amorphous chitin (with 44% DDA) are reported to show solubility in acidic solutions^24^. Chitin and chitosan have a range of uses in crucial areas such as, medical, agriculture, food/feed, cosmetic industries, paper industry, pharmacy, wastewater treatment and textiles. Besides these, chitin and its derivatives, mainly chitosan, have various useful biological properties: biocompatible, biodegradable, antimicrobial, wound healing and haemostatic^25–27^.

The extraction of chitin usually flows through two stages; demineralization using HCl to remove calcium carbonate and deproteinization using a NaOH solution to remove protein. Several techniques to extract chitin from different sources have been published^28–30^. The most common method for chemically isolating chitin from crustacean shells involves a number of major steps: the washing, grinding and sieving of raw shells, followed by their demineralization (elimination of calcium carbonate in dilute acidic acid). This is followed by deproteinization in aqueous NaOH or KOH. The use of enzymatic hydrolysis for deproteinization and microorganisms for both demineralization and deproteinization have been reported^31^. The cumbersome procedure involved and the byproducts released during each stage of interaction are challenges facing chitosan extraction processes. Also, the high susceptibility of chitosan to processing conditions (such as heating or freezing) can impose stress on its structure and cause polymer degradation^32^. Hence, newer methods and techniques have also been attempted, with the most efficient one being the microwave assisted extraction methodology^33–35^. Chitosan is a hot topic biomaterial that has excellent properties, but a global problem is that it is difficult to get a continuous and easily accessible supply.

In the current work, a novel method for obtaining chitosan nanofibrils from commercially available chitin using a one pot strategy involving graviola extract is demonstrated. Graviola extracts have been reported to interact with chitin and result in the breakdown of chitin when used as an insecticide^36^. This formed the foundation for the current study, where we allowed chitin and graviola extract to interact and examined the recovered product. In addition, graviola extracts were allowed to interact with shrimp, crab, and squid solid wastes and their final products characterized. This is the first report in this direction.

## Results and Discussion

### Chitosan nanofibers from commercial chitin

#### Morphological characterization

Acetogenin class of polyethers are found in the Annonaceae family of plants^37–39^. They consist of C32 or C34 fatty acid chains with terminal γ-lactones, additionally featuring, epoxide, hydroxyl, ketone, tetrahydrofuran and tetrahydropyran groups. Graviola belongs to the Annonaceae family, known for its acetogenins that possess anticancer activity. Acetogenins are also known for their dechitinization activity, which has formed the basis for their use against larvae of pests^40^. It is with this background, that the possibility for a positive interaction between chitin and graviola extract was conceived.

**Figure 1:**
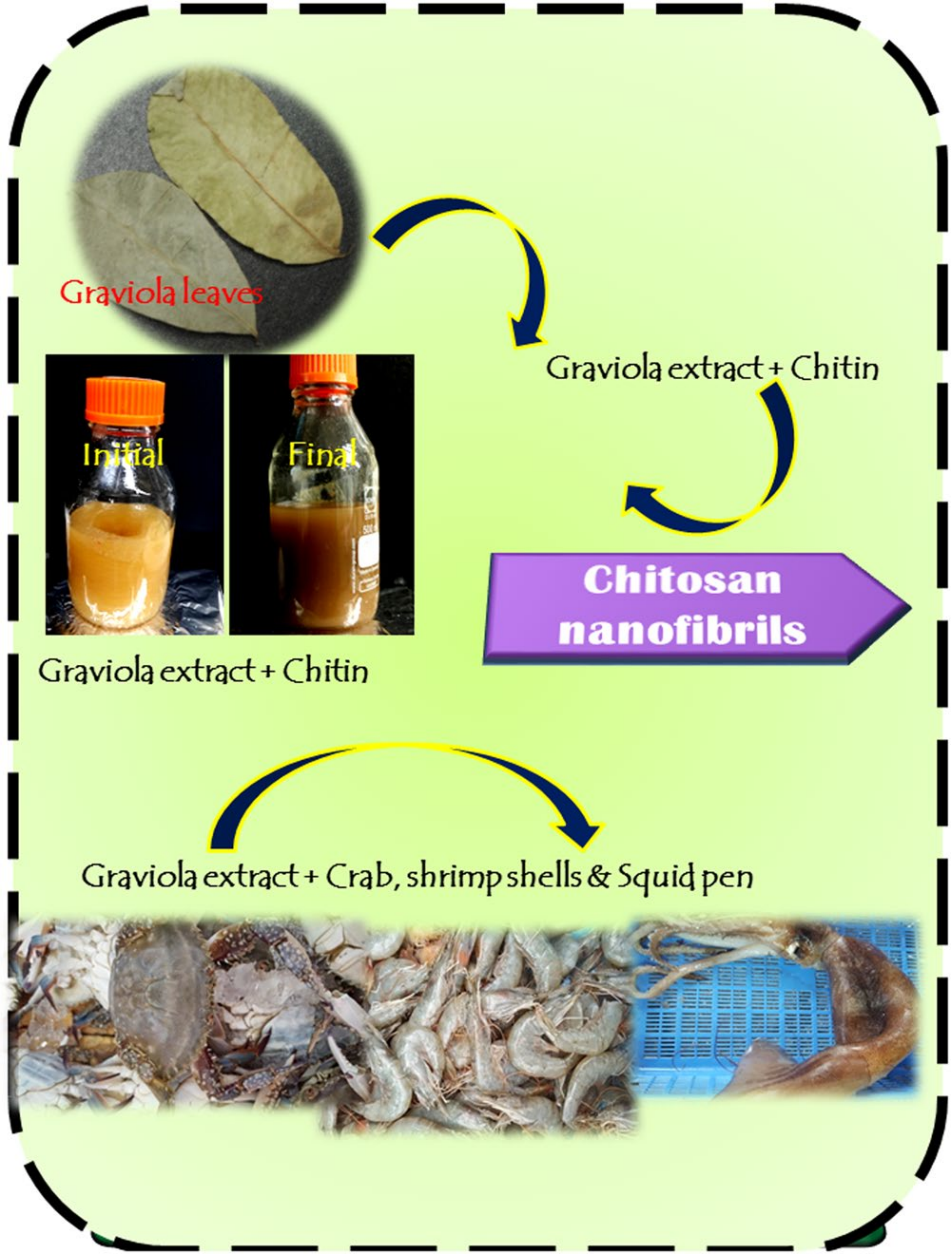
Schematic work flow of the study.

**Figure 2:**
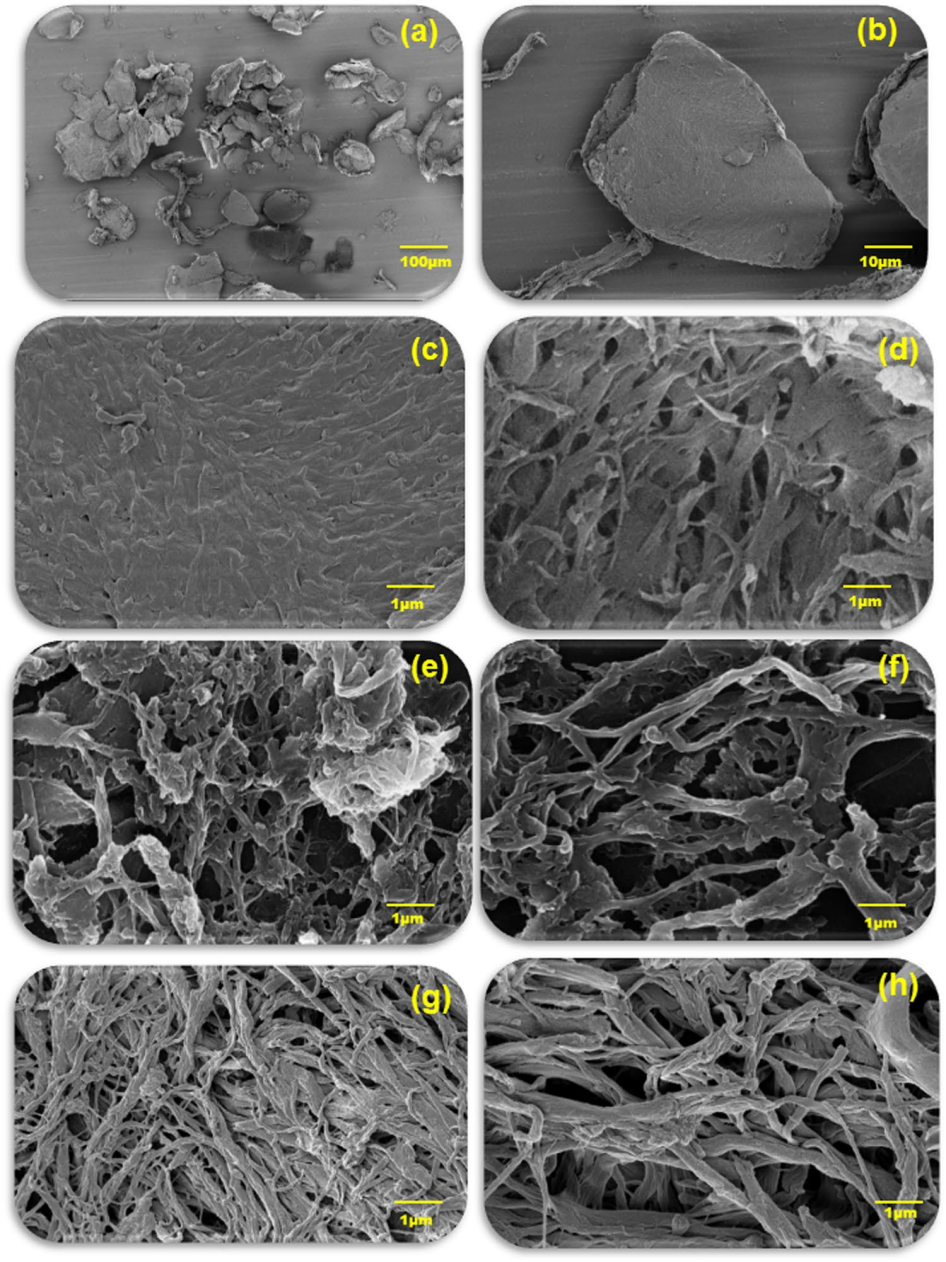
FESEM micrographs of (**a**,**b**) commercial chitin flakes incubated with GE for (**c**) 1 h (**d**) 1 day (**e**) 3days (**f**) 5 days (**g**) 6 days (**h**) 7 days.

**Figure 3:**
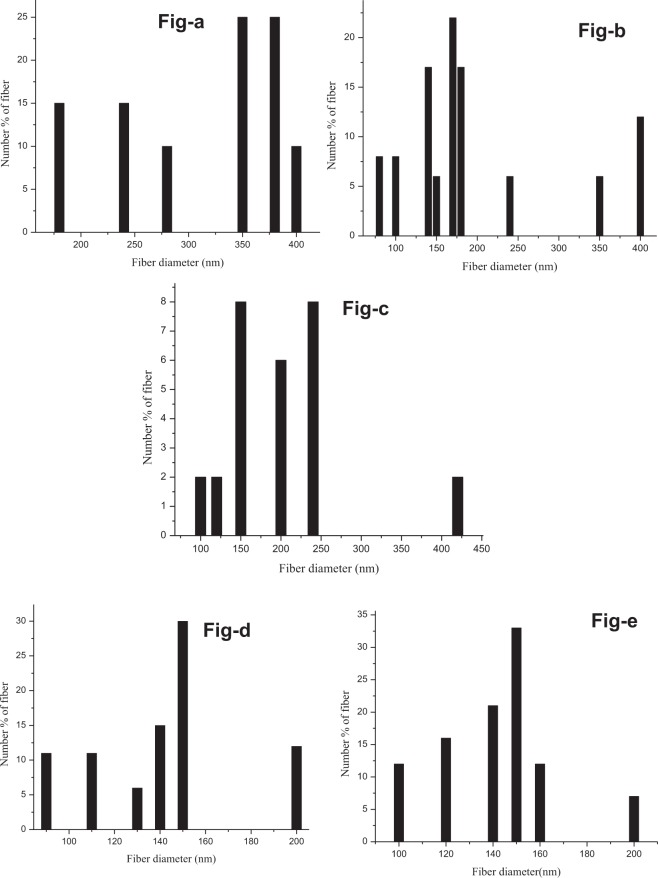
Size distribution histograms of chitosan nanofibers following incubation with GE for (**a**) 1 day (**b**) 3days (**c**) 5 days (**d**) 6 days (**e**) 7 days.

Commercial crab chitin was exposed to graviola extracts (GE) and samples retrieved after 1 day, 2 day, 3 day, 5 day, 6 day and 7 day intervals. Figure 1 displays the schematic of the work flow in this study. The morphology of the obtained material was imaged using FESEM. The FESEM results as observed in Fig. 2, reveals a progressive trend in the extraction of nanofibres from the source material, commercial crab chitin. The as-received chitin were microsized flakes (Fig. 2a–c), following interaction with GE after 1 day (Fig. 2d), 3 day (Fig. 2e), 5 day (Fig. 2f), 6 day (Fig. 2g) and 7 days (Fig. 2h) the nanofibres started showing up. Nanofibres in the size ranges upto 100–200 nm were obtained. The fibre sizes were measured using OPTIMAS software based on the FESEM images. Figure 3 gives the size distribution histogram of the fibres obtained after 1 day (Fig. 3a), 3 day (Fig. 3b), 5 day (Fig. 3c), 6 day (Fig. 3d) and 7 days (Fig. 3e). As observed from the histograms, fibres obtained @ 1 day were in the size range of 175–400 nm with sizes 350 nm to 375 nm predominating. As for fibres obtained @ 3 day exposure conditions (Fig. 3a), the size ranges were 75–400 nm with sizes 170 nm to 180 nm predominating (Fig. 3b). 5 day (Fig. 3c) exposure conditions resulted in sizes of 150 nm predominating; in 6 day (Fig. 3d) exposures, size ranges of 75–200 nm (140 nm predominating) and in 7 day (Fig. 3e) conditions, 100–150 nm sizes were observed. The dissolution of the protein matrix holding the chitin fibres, as a result of heat treatment, released the chitin fibrils. The size reduction of the fibres is due to the magnetic stirring effect^41^. The pH of the GE extract was 6.3 and remained unaltered throughout its interaction with the chitin material.

#### Chemical characterization

The chemical nature of the nanofibers is yet to be unverified, since it is known that chitin under specific conditions undergoes deacetylation to form chitosan. The nanofibers obtained from chitin, thus were characterized to ensure their chemical identity. Figure S1 gives the UV-Vis spectrum of the nanofibers in water. As observed from the figure, standard chitin shows absorption in the 100 to 250 nm range same as that of chitosan^42^. Spikes of varying intensity were observed in this range in the nanofibers harvested at varying interaction time from the GE extracts. The other peaks seen in the UV spectra are from components of the GE extract which show maximum absorption in the range of 200–230 nm. Based on the UV-Vis absorption peaks it is thus not definitive to conclude the nature of the nanofibres to be that of chitosan. But as observed from the spectra, it can be seen that the nanofibers also showed enhanced absorption in the 200–230 nm region compared to the standard chitin peaks, indicating functionalization of components from the extract on their surfaces. After an incubation period of 5 days a sudden spike in the chitin-chitosan absorption region was observed, which abruptly changed to a spike in the 220–230 nm region. Due to interference of the graviola acetogenin absorption (210–225 nm) lying in the same area as that of chitin and chitosan, not much conclusions could be arrived at using the UV-Vis results alone.

**Figure 4:**
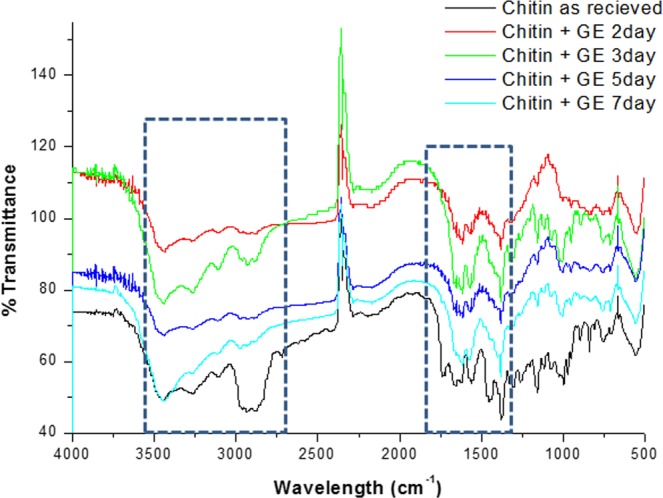
FTIR spectra of control chitin and GE interacted chitin.

FTIR is reportedly a more conclusive evidence, since it indicates the deacetylation of chitin clearly with marked changes in the FTIR bands. Figure 4 shows the results of the FTIR investigations, the peak at 3417 cm^−1^ was stronger due to the hydroxyl group superimposed on the amine group stretching vibrations. The peak at 2900 cm^−1^ is indicative of the CH bond^43^. Distinct changes in the 2900 cm^−1^ region are evident from Fig. 4. Compared to standard chitin, the GE interacted nanofibres show profound changes in this region indicating a transformation of chitin to chitosan. The 2 day @GE nanofibers show decrease in the 2900 cm^−1^ band, which continued in the 3 day@GE nanofibres and increased as a function of GE interaction upto 5 day GE nanofibres. Moreover, the other peaks at 1659 cm^−1^ and the two peaks at 1382 and 1247 cm^−1^ were caused by CO stretching mode of carboxyl group conjugated to a NH deformation mode resulting in CONH, indicative of amide bond formation. Following deacetylation of chitin major changes 1659 cm^−1^ undergoes changes and decays, the shape of bands between 1500 and 1750 cm^−1^ is changed too. Significant differences can also be observed in shape of 2800–3500 cm^−1^ band (Fig. 4) confirming the transformation of chitin to chitosan^44^. Figure S2 shows the FTIR bands of standard chitosan. DDA which is the confirmative test for ensuring chitosan’s identity was determined. As shown in Table 1, the DDA % obtained from the FTIR spectra of 2 day @ GE nanofibres, 3 day@ GE nanofibres, 5 day@ GE and 7 Day @GE, confirmed the chemical identity of the nanofibre as chitosan. The FTIR results thus conclusively confirmed the chemical identity of the obtained nanofibers to be that of chitosan and not chitin. Moreover, these results indicated that prolonged incubation did not yield better results, rather a drop in DDA was observed at 7 day @GE, hence 3 day to 5 day incubation are considered optimal. Yield of chitosan was calculated by comparing the weight of the raw material to the weight of chitosan, which was obtained after the treatment. The average yield % at 3 day and 5 day periods was 55.34% ± 5.57. In literature, different methods yielded varying results, based on the method, the extractant and the source material. Sarbon *et al*. claimed 44% from mud crab, Ibitoye *et al*., report 4–10% from house cricket^45,46^ and other authors report 72–75% from commercial chitin. A straightforward comparison within existing extraction methods and the current one could not be arrived at, since chitosan extraction is subject to a lot of variation.

**Table 1:** Degree of deacetylation of commercial chitin following interaction with GE.

Sample Name	Degree of deacetylation DDA %	Sample identity
Chitin + GE 1 day	50.97	Chitosan
Chitin + GE 3 day	94.86	Chitosan
Chitin + GE 5 day	92.73	Chitosan
Chitin + GE 7 day	86.37	Chitosan

### Chitosan from marine solid wastes

#### Morphological characterization

**Figure 5:**
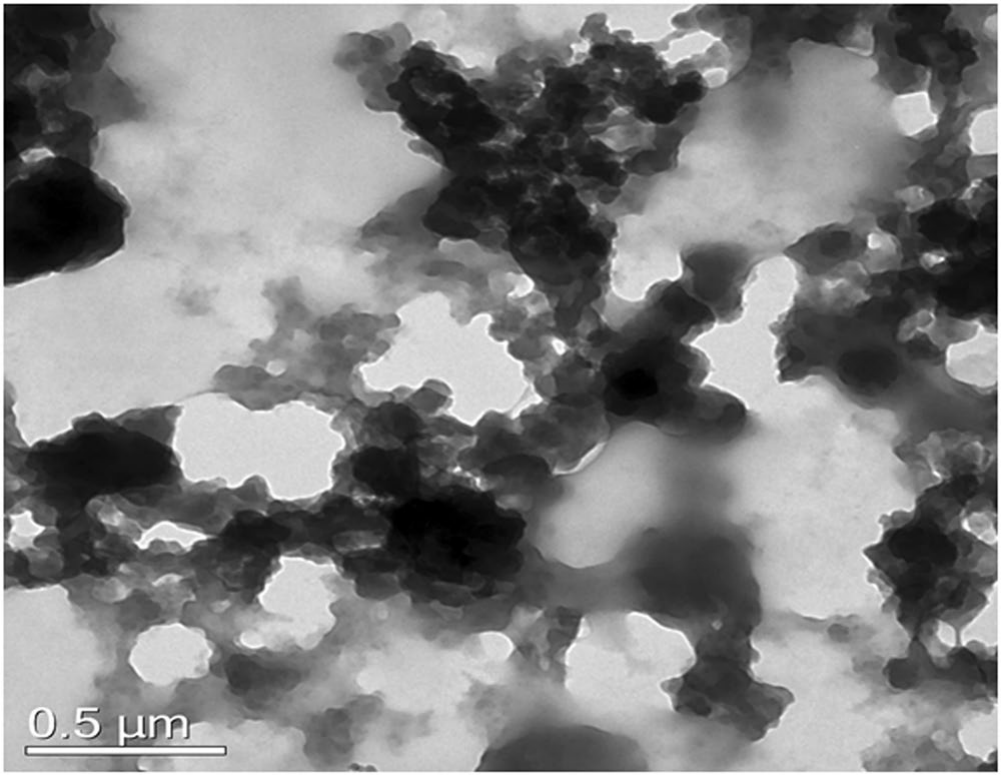
TEM of chitosan recovered from shrimp shells following GE interaction of 3 days.

Crab and shrimp solid shell wastes and squid pen were subjected to direct one pot GE interaction to assess the ability of GE towards recovery of chitin/chitosan directly from shells. Following incubation of 3 days with GE, the supernatant was tested for chitin/chitosan and the shells imaged using an inverted microscope. Fig. S3a–f summarizes these results. It was observed that visually the solid wastes turned black and became highly brittle in nature. Chitin being the structural framework of the exoskeleton, when removed into the medium, could result in this brittle condition. Figure 4a shows the nature of the crab shell prior to interaction with GE and Fig. 4b shows the shell morphology following interaction. The insets show their respective photographs. In case of crab not much difference was observed, but in case of shrimp (Fig. S3d) and squid pens (Fig. S3f) distinct changes in the morphology was observed. In case of shrimp evident signs of removal of structural components was observed, resulting in the voids observed in the image. As for squid pens (Fig. S3f), clear alterations in the striations was observed in the control or as received pens (Fig. S3e) were evident. As the insets confirm, visible changes could be seen prior to (Fig. S3a,c,e) and following GE interaction (Fig. S3b,d,e) with respect to the external morphology,

The supernatant was characterized for chitosan particles, Fig. 5 endorses the morphology of the obtained material. As observed from Fig. 5, the particles obtained following interaction with solid marine wastes did not possess the nanofibre morphology as observed in the above instance, but a colloidal particulate morphology.

#### Chemical characterization

The UV Vis spectrophotometric results as shown in Fig. S4, show absorption peaks indicative of chitin/chitosan in the 220–250 nm region. The peaks were highest in case of squid pens, followed by crab and shrimp shells. In α-chitin, all molecular chains are arranged in an antiparallel mode with strong intermolecular hydrogen bonding. β-chitin has a parallel chain packing with intermolecular forces weaker than those between chains of α-chitin^47^. This feature makes β-chitin more susceptible to enzymatic degradation or chemical reaction^48^. Most natural chitins have α-type structure, while β-type chitin is less diffuse^49^. Squid pens have β-type chitin and as it appears in our study too, they were the ones that enabled easy and effective recovery of chitin/chitosan as shown by the high absorbance compared to the shrimp and crab shells. The yield % was 22.34%, 34.44% and 18.21% for SS, SP and CS respectively.

**Figure 6:**
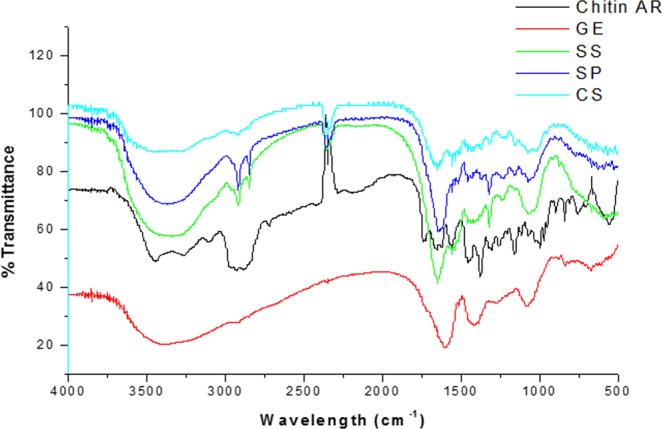
FTIR spectra of chitosan recovered from crab and shrimp shells and squid pen.

FTIR studies were undertaken and the results confirmed the recovery of chitosan from solid wastes. The recovered particles from the shells were confirmed to be that pertaining to chitosan and not chitin. Figure 6 clearly demonstrates characteristic chitosan bands on the FTIR spectra of the recovered material from crab shells, shrimp shells and squid pens. As shown in Table 2, the DDA % obtained from SS, SP and CS indicated the recovery of chitosan with 87%, 82% and 72% DDA respectively. Thus, it is confirmed that the recovered product from the marine solid wastes was not chitin but chitosan.

**Table 2:** Degree of deacetylation of marine solid wastes following interaction with GE.

Sample Name	Degree of deacetylation DDA %	Sample identity
Shrimp Shells (SS)	87.71	Chitosan
Squid Pens (SP)	82.11	Chitosan
Crab shell (CS)	72.73	Chitosan

**Figure 7:**
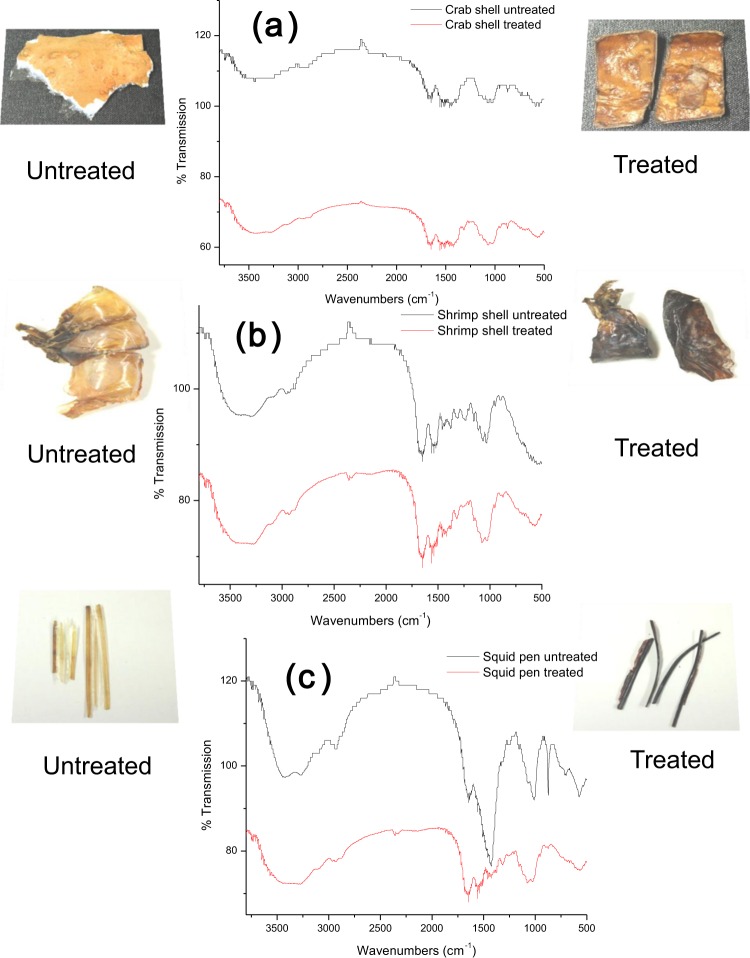
FTIR of (**a**) crab, (**b**) shrimp and (**c**) squid solid wastes, prior to treatment with GE and subsequent to GE exposure.

As a supporting evidence that the solid wastes on interaction with Graviola extracts did undergo chemical changes was confirmed by using FTIR to analyze the solid wastes of crab, shrimp and squid prior to treatment (untreated) and subsequent to interaction with GE. As clearly indicated in each of Fig. 7a–c from crab, shrimp and squid solid wastes, distinct decrease in % transmittance was observed following treatment. Decrease in absorption, signifies some changes (corresponding to the sample composition) related to the bonds or phase or crystallinity.

These results suggest that graviola extracts could orchestrate (i) recovery of nanofibres from commercial chitin material (ii) deacetylation of chitin to form chitosan and (iii) succeed in the recovery of chitosan from crude chitin from solid wastes of shrimp, crab and squids. This is the first report in this direction. The conventional extraction of chitin from shells comprised of stages; the first is demineralization using HCl to remove calcium carbonate and the second step being deproteinization using a NaOH solution to remove protein. To obtain chitosan a further deacetylation protocol is mandatory. The demonstrated methodology is completely green and is orchestrated solely by the plant extract interaction.

## Discussion

The idea of using graviola extracts which were known for their anticancer activity for chitosan recovery started with the disclosure of its ability to act as an insecticide. Graviola extracts could lead to disruption of egg shells and dechitinization of insect/larvae body walls, as reported^50^. This brewed the idea of a possible interaction between the graviola extract and chitin which conceived this pioneering work. The one pot, go green, protocol resulted in the retrieval of chitosan from commercial chitin and also directly from solid marine wastes. Graviola, commonly called soursop belongs to the Annonaceae family, whose scientific name is *Annona muricata*. Graviola plays a crucial role in various traditional and alternative medicines. All parts of this evergreen tree inhabiting tropical and sub tropical areas are used in natural medicine. Graviola extracts are known to be rich in flavonoids, isoquinoline alkaloids and annonaceous acetogenins^51^. Acetamide, acetic acid, formamide, decanoic acids and its associates, xylitol, piperidine, propanoic, threonic, coumaric, xylonic, lactic, maleic acids and various bioactive sugars have been identified in the extract. It is also well known that the presence of alkaloids, flavonoids, terpenoids, coumarins and lactones, anthraquinones, tannins, cardiacglycosides, phenols, phytosterols, and saponins validate the economic importance of *A*. *muricata* leaves extracts for extensive use in medicine both traditionally and pharmaceutically.

### Mechanism of action of graviola extracts

**Figure 8:**
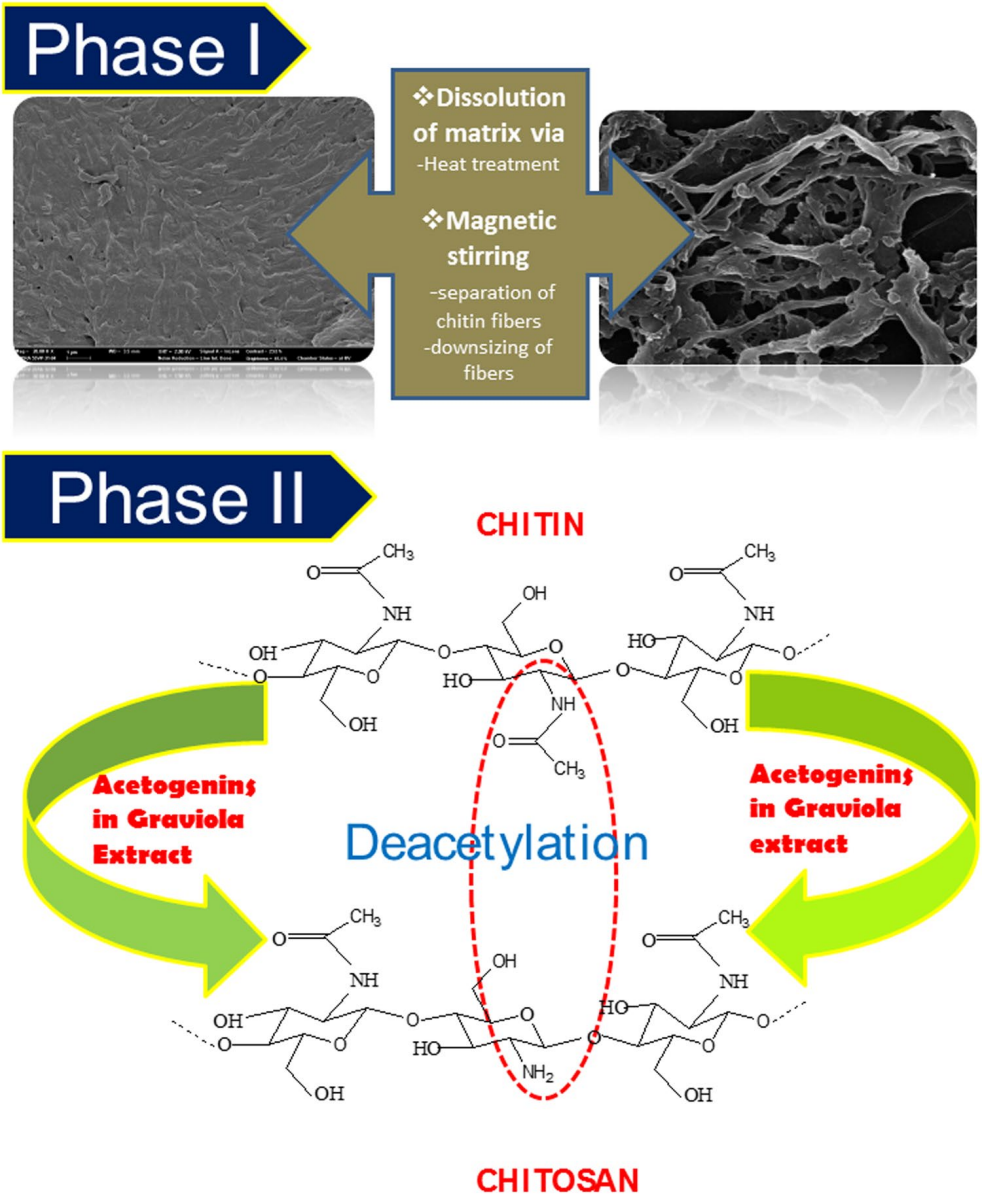
Speculated mechanism orchestrated by GE for the deacetylation of chitin to chitosan.

**Figure 9:**
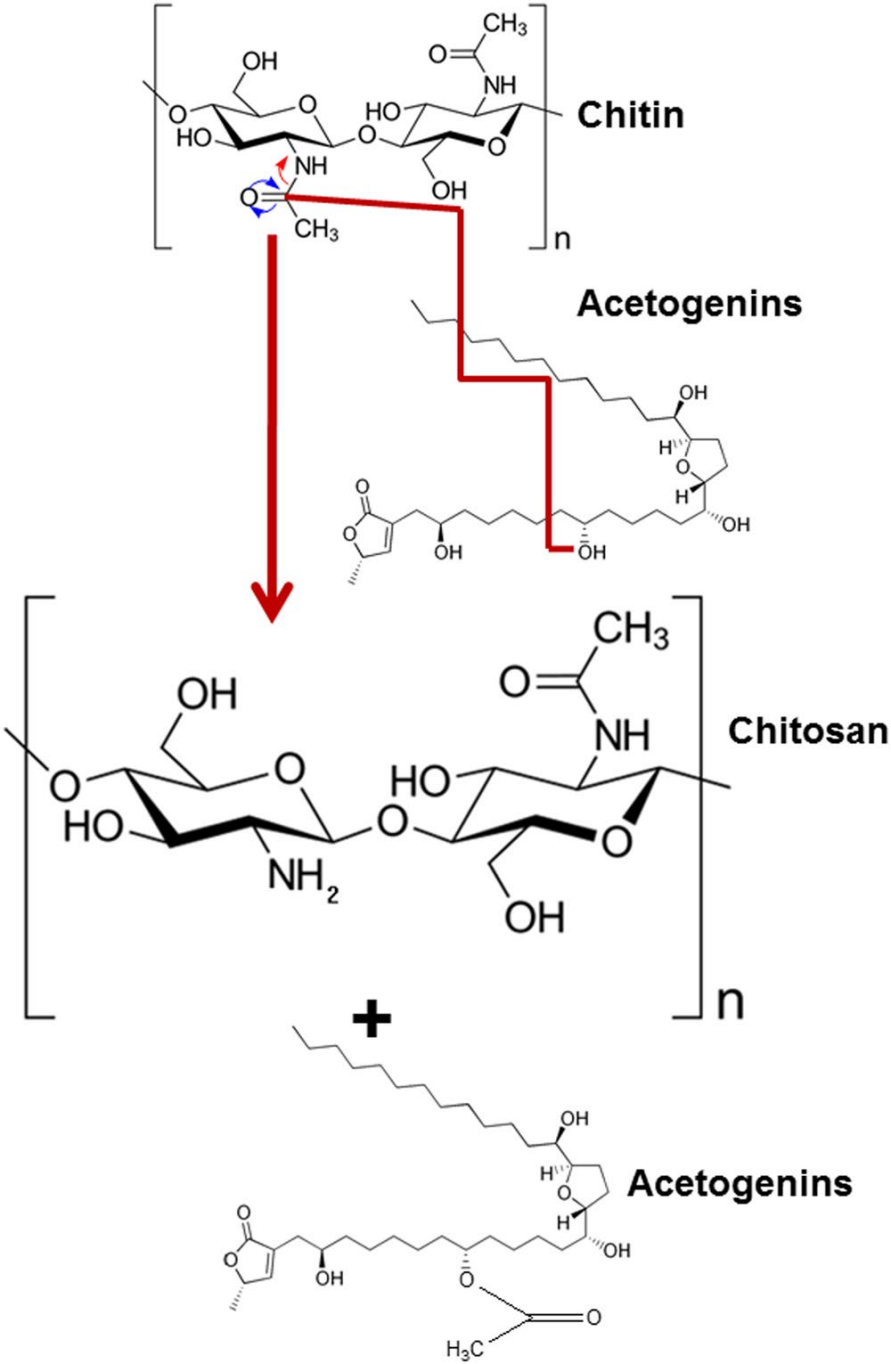
Illustration of the deacetylation reaction steered by the acetogenins in the graviola extracts.

The interaction of chitin with GE resulted in chitosan nanofibers from commercial chitin flakes and chitosan particles from solid shell wastes and squid pens. Graviola extract is a rich reservoir of various groups of compounds. Graviola extracts are well established as a source of anticancer drugs and for its bioactive potential, till date no reports exist on the use of Graviola for green synthesis or phytosynthesis of nanomaterials. Figure 8 speculates the possible mode of action involved in the transition of chitin to chitosan. A physio-chemical operation is believed to be the basis. The heating of the interactants resulted in the dissolution of the protein matrix, releasing the chitin fibers. Magnetic stirring resulted in the splitting up and smoothening of the fibre aggregates. The exposed fibers were now available to freely interact with the components in the graviola extract, orchestrating the deacetylation of chitin to form chitosan. In case of the solid shells and squid pens, deproteinization through heating and dissolution of chitin into the extracts and their subsequent deacetylation is the operational strategy. The chitosan recovered from the shells did not possess a fiber-based morphology but formed powders made of particulate chitosan. The UV Vis spectra showed that the absorbance of the nanofibers revealed the presence of the acetogenins. The adsorption of acetogenins on chitosan nanofibers point towards the direction of an acetogenin lead deacetylation mechanism operating. Deacetylation reaction of chitin is probably facilitated by the nucleophilic attack of OH groups present in the acetogenins (Fig. 9). More conclusive studies will shed light on the exact modus operandi involved.

Graviola (soursop), Annona muricata L., is widely cultivated throughout tropical regions of the world, and occurs as a small tree. The fruits are that which are edible, the leaves were considered insignificant till their anticancer value was unearthed. With the wide abundance of this plant and with the fact that we do not use the roots or the entire plant, there is no question about the sustainable harvesting of its leaves in event of a scale-up for industrial large scale chitosan recovery. The occurrence of this plant, wild and cultivated and not endangered are additional stilts that keep the sustainability aspect strong.

## Conclusion

For the first time the interaction between graviola extracts and chitin were studied. As hypothesized the results confirmed graviola mediated one pot recovery of chitosan from chitin and solid shell wastes. This study pioneers the hope for cheap and sustainable recovery of chitosan (reputed for its enormous biological applications) enabled through this phyto mediated green technique. Further, the graviola extraction process by itself is obtained without any chemical intervention, solely via a hot water based extraction methodology.

## Methods

### Chitin sources

The chitin used for the preparation of chitosan nanofibrils was commercial chitin from crab shells, practical grade (C7170-100G) from SIGMA-ALDRICH, Inc., 3050 Spruce Street, Louis. MO 63103 USA 314-771-5765. The crab and shrimp shells and squid pens were obtained from a local restaurant in Gwanjin gu, Seoul. South Korea. The dried Graviola leaves used for preparing the extract were procured from a traditional medicine outlet in Seoul, Korea. All the chemicals used in the study, unless specified otherwise, were all of analytical grade. Millipore water was used for all experiments. All methods were carried out in accordance with relevant guidelines and regulations. No live animals were used in the study. Only waste marine shells collected from restaurants were used.

### Graviola extract

Four grams of dry leaves (~8 leaves) were cut into small strips and used for preparing the extracts. A co-boiling water extraction methodology was practiced, since our previous experiments^52^ had shown that co-boiling method was better than the green tea style (preboiling water and then adding leaves for extraction process, off flame). The leaves were added to 200 mL sterile water in a flask and the contents boiled until evaporated to half the original volume. The extract pH was measured and used in the ensuing studies. The extract was coded GE.

### Chitosan recovery from commercial chitin

Two grams of commercial crab shell chitin (CC) was added to 200 mL of GE and allowed to interact on a magnetic stirrer cum heater, set at 70 °C for 1, 2, 3, 4, 5, 7 days. The retrieved samples were centrifuged (Beckman Coulter Avanti J-25, USA) at 3000 rpm for 3 min to pellet the bigger debris. The pellet was discarded and the supernatant was centrifuged at 19,000 rpm for 15 min and the pellet was suspended in distilled water. One set of the pellet was dried and used for FTIR analysis.

### Chitosan recovery from crab-shrimp-pen wastes

One gram of crab and shrimp shells and 0.5 g squid pens were surface sterilized in 70% ethanol for 5 min and then dried in a laminar air flow under sterile conditions. One inch sized pieces were cut and incubated with 200 mL of GE and allowed to interact on a magnetic stirrer cum heater, set at 70 °C for 3 days. The supernatant was collected in centrifuge tubes and pelleted (Beckman Coulter Avanti J-25, USA) at 19,000 rpm for 15 min. The pellet was stored upto further use. One set of the pellet was dried and used for FTIR analysis. Figure S1 gives the schematic work flow.

### Characterization of chitosan

The suspended pellet following GE incubation for varying time periods was characterized using a Nanodrop ND-1000 v 3.3.1 spectrophotometer, (Nanodrop Technologies, Inc., Wilmington, USA). The absorbance was scanned from 220–700 nm. Fourier-transform infrared spectroscopy (FTIR) (Shimadzu FTIR-8300 spectrometer, San Diego, CA, USA) of the recovered product and of the solid wastes prior to and subsequent to treatment was done using KBr pellets. Field emission scanning electron microscopy (FE-SEM) (JEOL, JSM-5410LV) was used to image the synthesized product. The morphological changes on the shells and squid pens prior to and subsequent to incubation with the extract were imaged using an inverted microscope, Axiovert 2000, Carl Zeiss, Germany. The chitosan recovered from interaction with marine shell waste was characterized using a JEM-1400PLUS, transmission electron microscope (TEM), JEOL USA, Inc. Peabody, MA, USA. For calculating the degree of deacetylation (DDA) to confirm the chemical identity of chitosan using FTIR spectra, several procedures and equations are described in literature^53,54^. These equations were derived on the basis of calibration curves, and the calculation procedures are based on absorbance ratios of various spectral bands^55,56^. The equation^57^ used in this study is listed below.

$$\mathrm{DA}[ \% ]={{\rm{A}}}_{1655}/{{\rm{A}}}_{3450}\times 115$$

## Supplementary information

Supporting Info

## References

[1] Connors M (2012). Three-dimensional Structure of the Shell Plates Assembly of the Chiton *Tonicella marmorea* and Its Biomechanical Consequences. J. Struct. Biol..

[2] Majtan J (2007). Isolation and characterization of chitin from bumblebee (*Bombus terrestris*). Int. J. Biol. Macromol.

[3] Ai H, Wang F, Yang Q, Zhu F, Lei C (2008). Preparation and biological activities of chitosan from the larvae of housefly, *Musca domestica*. Carbohydr. Polym..

[4] Ifuku S, Nomura R, Morimoto M, Saimoto H (2011). Preparation of Chitin Nanofibers from Mushrooms. Materials.

[5] Marzia Bo (2012). Isolation and identification of chitin in the black coral *Parantipathes larix* (Anthozoa: Cnidaria) Inter. J Biol Macromol.

[6] Klinger C (2019). Express method for isolation of ready-to-use 3D chitin scaffolds from A*plysina archeri* (aplysineidae: verongiida) demosponge. Mar. Drugs.

[7] Shaala LA (2019). New Source of 3D Chitin Scaffolds: The Red Sea Demosponge *Pseudoceratina arabica* (Pseudoceratinidae, Verongiida). Mar. Drugs.

[8] Zóltowska-Aksamitowska S (2018). The demosponge Pseudoceratina purpurea as a new source of fibrous chitin. Inter. J Biol Macromol.

[9] Bechmann Nicole, Ehrlich Hermann, Eisenhofer Graeme, Ehrlich Andre, Meschke Stephan, Ziegler Christian, Bornstein Stefan (2018). Anti-Tumorigenic and Anti-Metastatic Activity of the Sponge-Derived Marine Drugs Aeroplysinin-1 and Isofistularin-3 against Pheochromocytoma In Vitro. Marine Drugs.

[10] Azizur Rahman, M. & Halfar, J. First evidence of chitin in calcified coralline algae: new insights into the calcification process of Clathromorphum compactum. *Scientific Reports* **4**, 61629 (2014). 10.1038/srep06162 PMC4141250 25145331

[11] Brunner, E. *et al*. Chitin-based organic networks – an integral part of cell wall biosilica from the diatom Thalassiosira pseudonana’. *Angew Chem Int Ed* **48**, 9724–9727 (2009a). 10.1002/anie.200905028 19924754

[12] Rinaudo M (2006). Chitin and chitosan: Properties and application. Prog. Polym. Sci..

[13] Marcin Wysokowski (2014). Identification of chitin in 200-million-year-old gastropod egg capsules. Paleobiology.

[14] Austin PR (1988). Chitin solutions and purification of chitin. Methods Enzymol..

[15] Roberts, G. A. F. *Chitin Chemistry*, 1st ed.; MacMillan: London, UK (1992).

[16] Jang MK (2004). Physicochemical characterization of α‐chitin, β‐chitin, and γ‐chitin separated from natural resources. J. Polym Sci. A Polym. Chem..

[17] Campana-Filho SP (2007). Extraction, structures and properties of α- and β-chitin. Quim. Nova.

[18] Kaya M (2014). Extraction and Characterization of α-Chitin and Chitosan from Six Different Aquatic Invertebrates. Food Biophysics.

[19] Murat Kaya (2017). On chemistry of γ-chitin. Carbohydrate Polymers.

[20] Zhang Y, Zhang X, Ding R, Zhang J, Liu J (2011). Determination of the degree of deacetylation of chitosan by potentiometric titration preceded by enzymatic pretreatment. Carbohydr. Polym..

[21] Weinhold MX, Sauvageau JCM, Kumirska J, Thöming J (2009). Studies on acetylation patterns of different chitosan preparations. Carbohydr. Polym..

[22] Bobu E, Nicu R, Lupei M, Ciolacu FL, Desbrières J (2011). Synthesis and characterization of n-alkyl chitosan for papermaking applications. Cellul. Chem. Technol..

[23] Obaidat R (2010). Some physico-chemical properties of low molecular weight chitosans and their relationship to conformation in aqueous solution. J. Solution Chem..

[24] Smitha K.T., Anitha A., Furuike T., Tamura H., Nair Shantikumar V., Jayakumar R. (2013). In vitro evaluation of paclitaxel loaded amorphous chitin nanoparticles for colon cancer drug delivery. Colloids and Surfaces B: Biointerfaces.

[25] Puvvada YS, Vankayalapati S, Sukhavasi S (2012). Extraction of chitin from chitosan from exoskeleton of shrimp for application in the pharmaceutical industry. Int. Curr. Pharm. J..

[26] Chellamani KP, Balaji RSV, Sudharsan J (2013). Chitosan treated textile substrates for wound care applications. J. Acad. Indus. Res..

[27] Li X, Nan K, Li L, Zhang Z, Chen H (2012). *In-vivo* evaluation of curcumin nanoformulation loaded methoxy poly(ethylene glycol)-graft-chitosan composite film for wound healing application. Carbohydr. Polym..

[28] Al Sagheer FA, Al-Sughayer MA, Muslim S, Elsabee MZ (2009). Extraction and characterization of chitin and chitosan from marine sources in Arabian Gulf. Carbohydr. Polym..

[29] Synowiecki J, Al-Khateeb NA (2003). Production, Properties, and Some New Applications of Chitin and Its Derivatives. Crit. Rev. Food Sci. Nutr..

[30] Hayes M, Carney B, Slater J, Brück W (2008). Mining marine shellfish wastes for bioactive molecules: Chitin and chitosan -Part A: Extraction methods. Biotechnol. J..

[31] Struszczyk MH (2002). Chitin and Chitosan: Part I. Properties and production. Polimery.

[32] Szymańska E, Winnicka K (2015). Stability of chitosan-a challenge for pharmaceutical and biomedical applications. Mar Drugs..

[33] Samar, M. M., El-Kalyoubi, M. H., Khalaf, M. M. & El-Razik, A. M. M. Physicochemical, Functional, Antioxidant and Antibacterial Properties of Chitosan Extracted From Shrimp Wastes by Microwave Technique. *Annals of Agricultural Science* **58**, pp. 33–41 (2013).

[34] H.El KnidriR.El KhalfaouyA.LaajebA.AddaouA.Lahsini Eco-friendly extraction and characterization of chitin and chitosan from the shrimp shell waste via microwave irradiation Process Safety and Environmental Protection Volume 104, Part A, November, Pages 395–405 (2016).

[35] Sebastian J (2019). Microwave-assisted extraction of chitosan from Rhizopus oryzae NRRL 1526 biomass. Carbohydrate Polymers.

[36] Moghadamtousi Soheil, Fadaeinasab Mehran, Nikzad Sonia, Mohan Gokula, Ali Hapipah, Kadir Habsah (2015). Annona muricata (Annonaceae): A Review of Its Traditional Uses, Isolated Acetogenins and Biological Activities. International Journal of Molecular Sciences.

[37] Priya, V., Jananie, R. K. & Vijayalaxmi, K. GC MS determination of bioactive components in Pleurotus ostreatus Int Res. *J. Pharm.,* **3**, 150–151 (2012).

[38] Kim CS, Oh E-T, Lim D-H, Lim D-S, Keum Y-S (2015). Profiles of alkylresorcinols in Iris plants. Biochem. Syst. Ecol..

[39] Rupprecht JK, Hui YH, McLaughlin JL (1990). Annonaceous acetogenins: a review. J Nat Prod., Mar-Apr.

[40] Guadaño A, Gutiérrez C, De La Peña E, Cortes D, González-Coloma A (2000). Insecticidal and Mutagenic Evaluation of Two Annonaceous Acetogenins. Journal of Natural Product.

[41] Sun S (2007). Synthesis and characterization of biocompatible Fe3O4 nanoparticles. Journal of Biomedical Materials Research A.

[42] Harmoudi H (2014). Removal of 2,4-D from aqueous solutions by adsorption processes using two biopolymers: chitin and chitosan and their optical properties. Optical Materials.

[43] Abdou ES, Nagy KS, Elsabee MZ (2008). Extraction and characterization of chitin and chitosan from local sources. Bioresour. Technol..

[44] Sagheer F, Al-Sughayer M, Muslim S, Elsabee MZ (2009). Extraction and characterization of chitin and chitosan from marine sources in Arabian Gulf. Carbohydr. Polym..

[45] Sarbon NM (2015). Chitosan Extracted from Mud Crab (Scylla Olivicea) Shells: Physicochemical and Antioxidant Properties. Journal of Food Science and Technology.

[46] Ibitoye EB (2018). Extraction and physicochemical characterization of chitin and chitosan isolated from house cricket. Biomed Mater..

[47] Ianiro A (2014). Customizing Properties of β-Chitin in Squid Pen (Gladius) by Chemical Treatments. Mar. Drugs.

[48] Minke R, Blackwell J (1978). The structure of alpha-chitin. J. Mol. Biol..

[49] Moghadamtousi SZ (2015). Annona Muricata (Annonaceae): A Review of Its Traditional Uses, Isolated Acetogenins and Biological Activities Ed. Maurizio Battino. International Journal of Molecular Sciences.

[50] Gavamukulya Y, Abou-Elella F, Wamunyokoli F, AEl-Shemy H (2014). Phytochemical screening, anti-oxidant activity and *in vitro* anticancer potential of ethanolic and water leaves extracts of Annona muricata (graviola). Asian Pac J Trop Dis..

[51] Paul J, Gnanam R, Jayadeepa RM, Arul L (2013). Anti cancer activity on graviola, an exciting medicinal plant extract vs various cancer cell lines and a detailed computational study on its potent anti-cancerous leads. Curr. Top. Med. Chem..

[52] Chun Se, Xiaomin Shang, Anthonydhason Vimala, Jung Hyejin, Tilahun Belachew Shimels, Gopal Judy, Paul Diby (2018). Enhanced Harnessing of the Graviola Bioactive Components Using a Neoteric Sonication Cum Microwave Coadjuvant Extraction Protocol. Applied Sciences.

[53] Dong Y (2001). Determination of degree of substitution for N-acylated chitosan using IR spectra. Sc. China Ser. B-Chem..

[54] Czechowska-Biskup R, Jarosińska D, Rokita B, Ulański P, Rosiak JM (2012). Determination of degree of deacetylation of chitosan - comparision of methods. Prog. Chem. Appl. Chitin Its Deriv..

[55] Duarte M, Ferreira M, Marväo M, Rocha J (2002). An optimized method to determine the degree of acetylation of chitin and chitosan by FTIR spectroscopy. Int J Biol Macromol.

[56] Brugnerotto J (2001). An infrared investigation in relation with chitin and chitosan characterization. Polymer.

[57] Kasaai M (2008). A review of several reported procedures to determine the degree of N-acetylation for chitin and chitosan using infrared spectroscopy. Carbohydr Polym.

