# Supplementary material for: Sustainable ecofriendly phytoextract mediated one pot green recovery of chitosan
Source: Sci Rep. 2019 Sep 25;9:13832. doi: 10.1038/s41598-019-50133-z (PMC6761131; doi:10.1038/s41598-019-50133-z)
Supplement: Supplementary file 1 — Supporting Info [file 41598_2019_50133_MOESM1_ESM.pdf]

Sustainable ecofriendly phytoextract mediated one pot green recovery of chitosan

Judy Gopal[1±], Manikandan Muthu[1±], Thirumalai D[2], Ki Jun Kim[3], Nazim Hasan[4], Seong Jung Kwon[3] and Sechul Chun[1*]

[1]Department of Environmental Health Science, Konkuk University, Seoul 143-701, Korea.

[2]Department of Chemistry, Thiruvalluvar University, Vellore, Tamil Nadu, India 632115

[3]Department of Chemistry, Konkuk University, Gwangjin-gu, Seoul, Korea

[4]Department of Chemistry, Faculty of Science, Jazan University, Jazan, P.O. Box 114, KSA

[*] Corresponding author: Tel.: +8224500574; Fax: +8224503310

e-mail: scchun@konkuk.ac.kr

± Co-first authors: both authors contributed equally towards this manuscript

Supporting Fig Captions

Fig. S1 UV –Vis spectra of control chitin and GE interacted chitin

Fig. S2 FTIR of commercial available chitosan standard for reference

Fig. S3 Optical micrographs of crab shells (a) before and (b) after GE interaction; shrimp shells (c) before and (d) after GE interaction and squid pen (e) before and (f) after GE interaction. Inset show photographs of the external morphology of the respective optical micrographs.

Fig S4 UV –Vis spectra of chitosan recovered from crab and shrimp shells and squid pens

Fig. S1

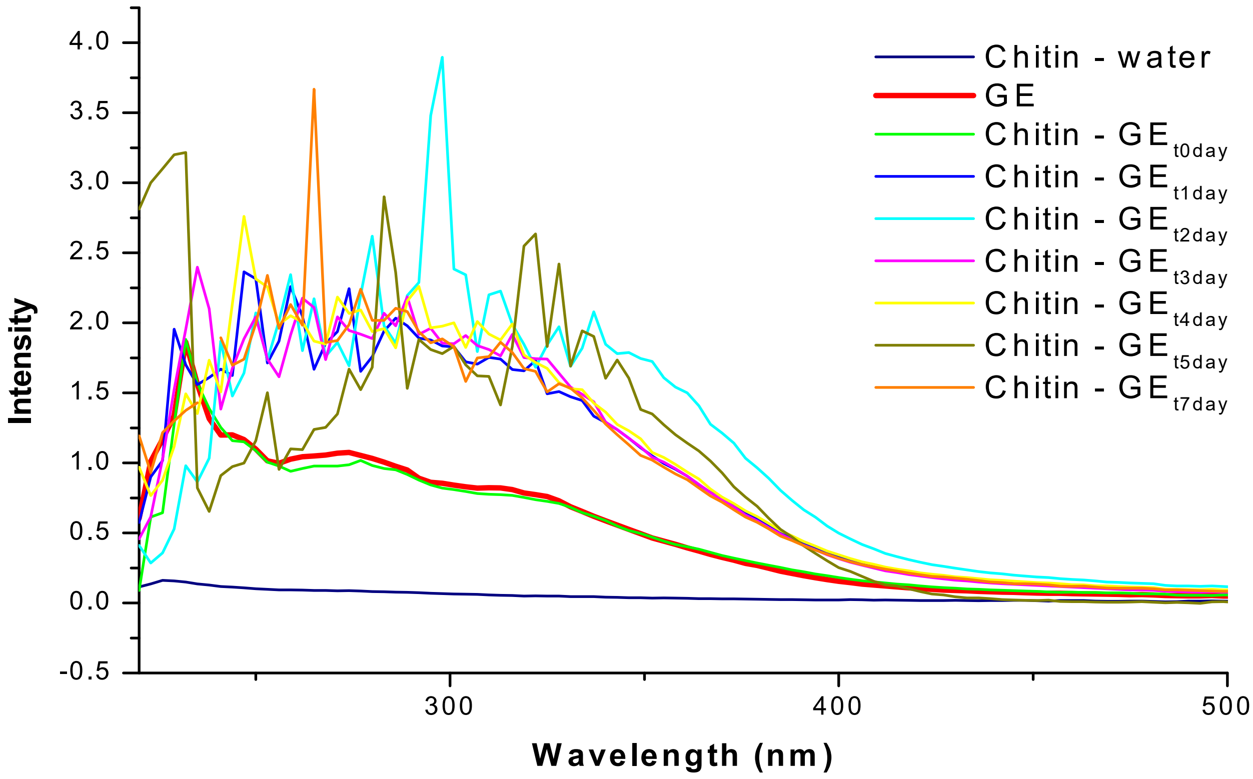
Intensity
Wavelength (nm)
4.0
3.5
3.0
2.5
2.0
1.5
1.0
0.5
0.0
-0.5
300
400
500
Chitin - water
GE
Chitin - GE t0day
Chitin - GE t1day
Chitin - GE t2day
Chitin - GE t3day
Chitin - GE t4day
Chitin - GE t5day
Chitin - GE t7day

Fig. S2

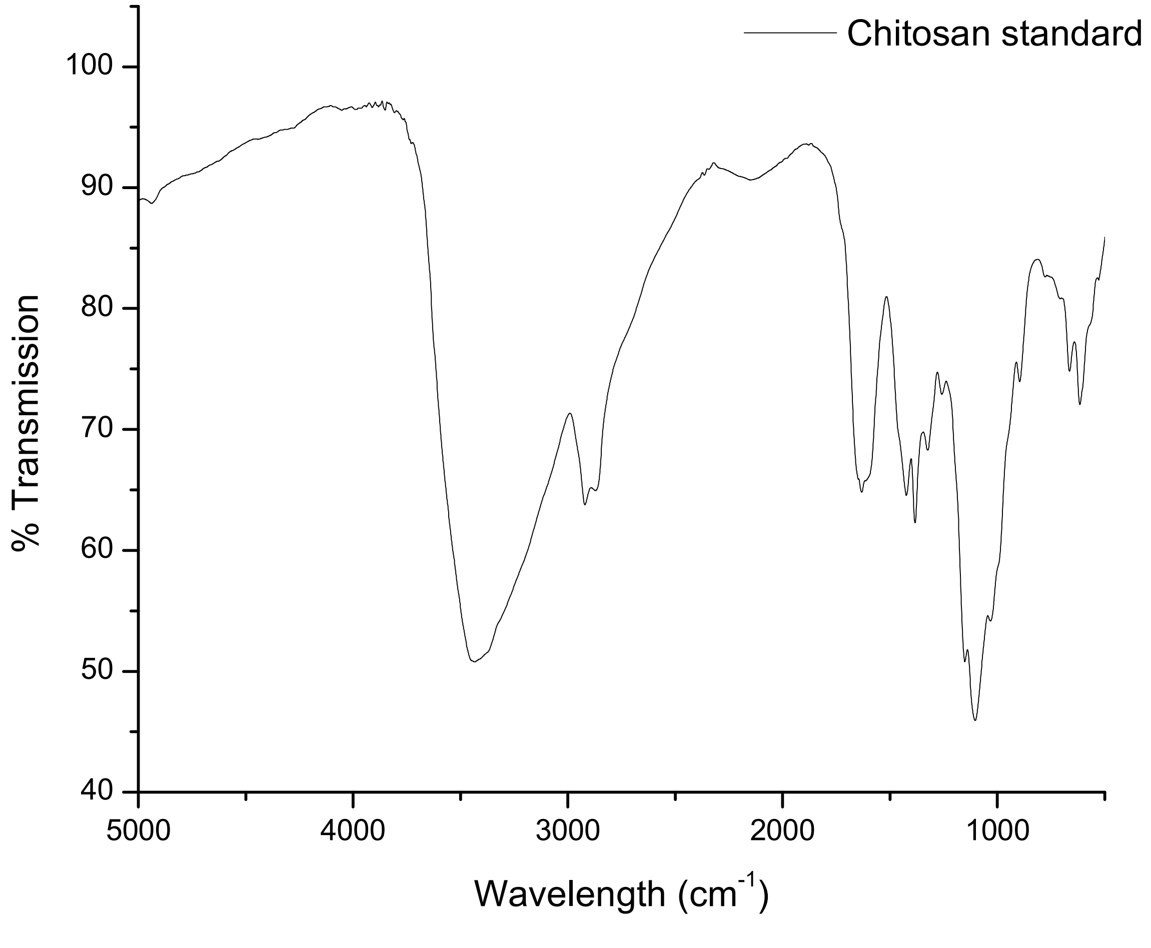
Chitosan standard
% Transmission
100
90
80
70
60
50
40
5000
4000
3000
2000
1000
Wavelength (cm-1)

Fig. S3

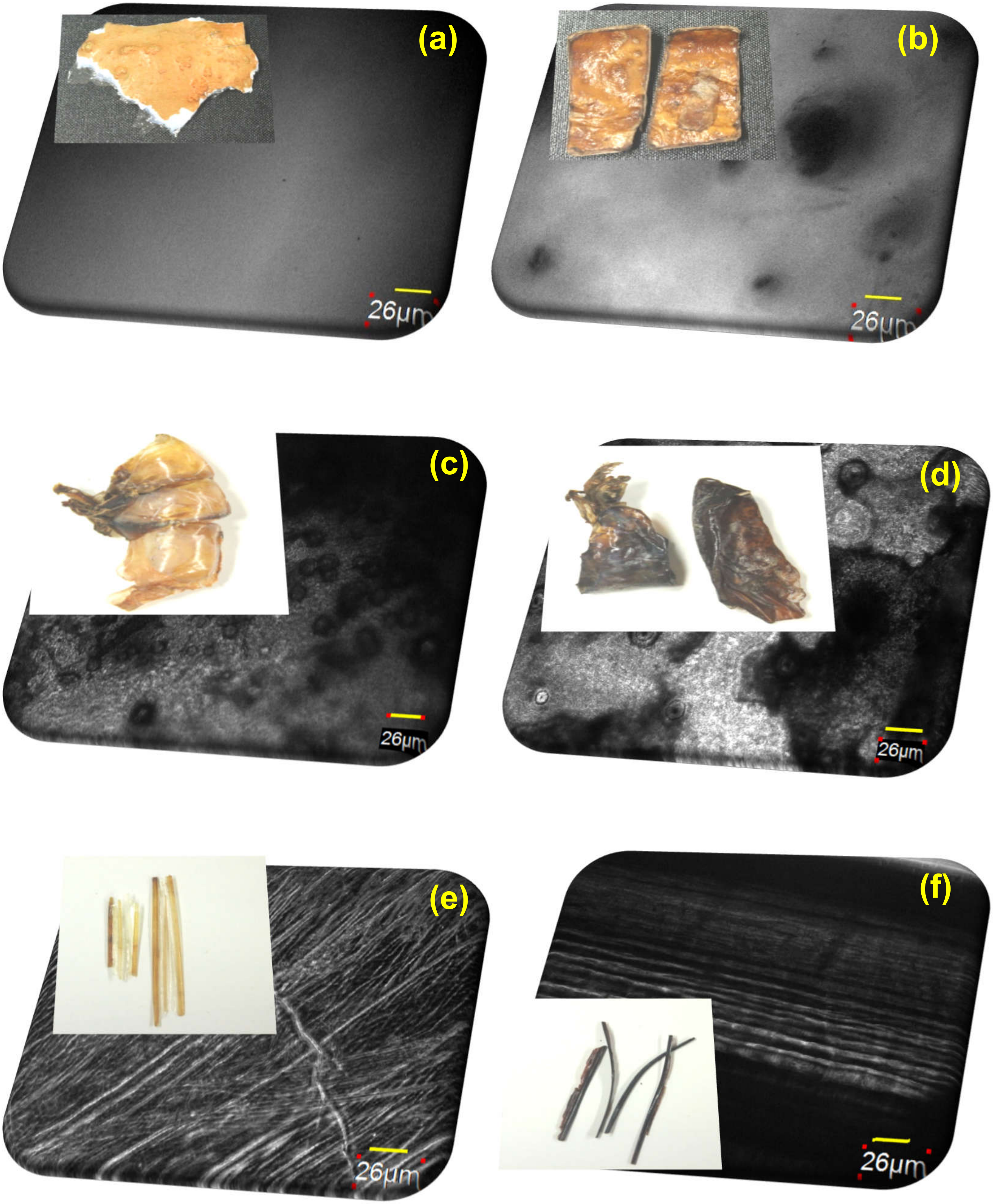

Fig. S4

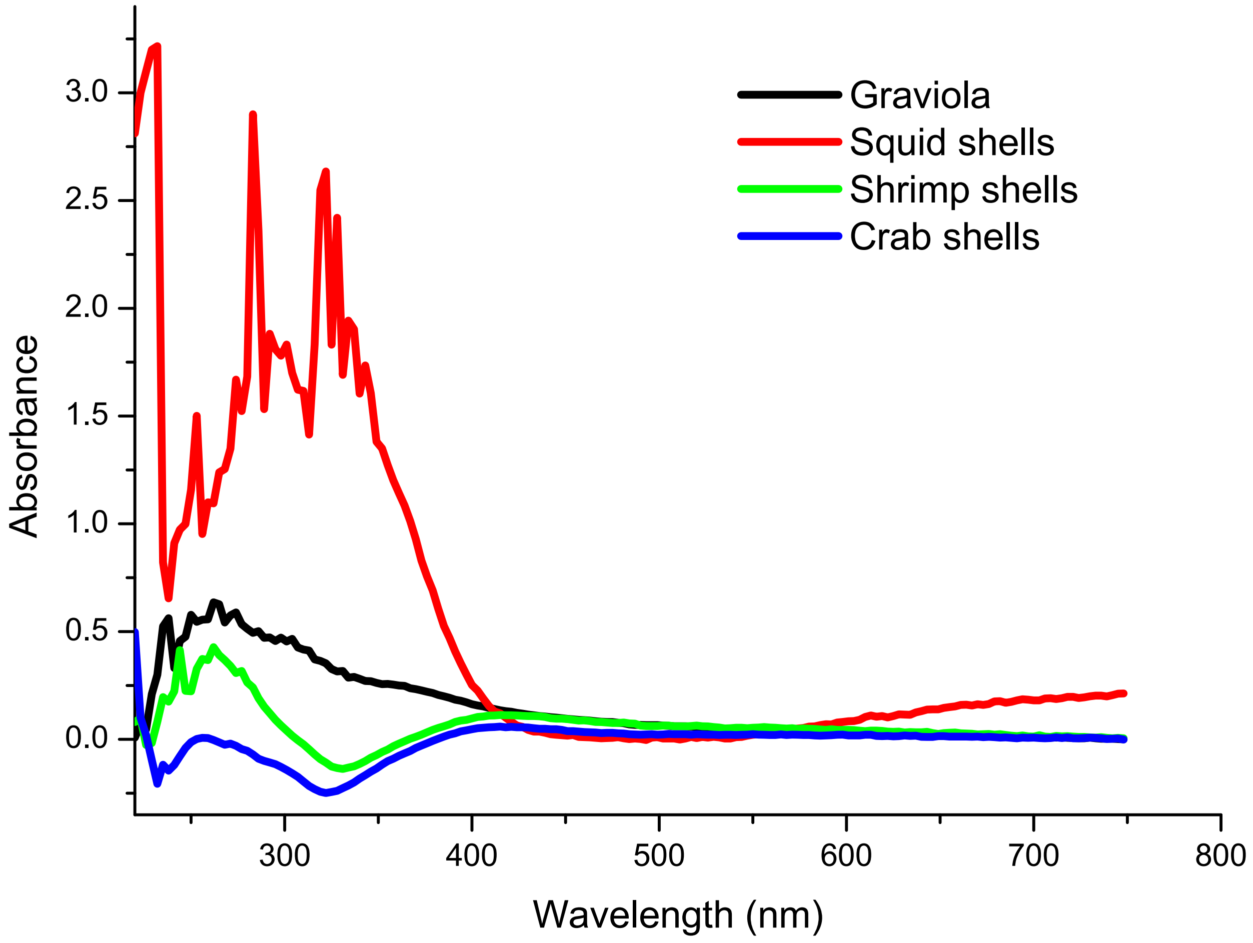

Graviola
Squid shells
Shrimp shells
Crab shells
Absorbance
Wavelength (nm)
0.0
0.5
1.0
1.5
2.0
2.5
3.0
300
400
500
600
700
800
